# Molecular docking and inhibition studies on the interactions of *Bacopa monnieri’*s potent phytochemicals against pathogenic *Staphylococcus aureus*

**DOI:** 10.1186/s40199-015-0106-9

**Published:** 2015-04-17

**Authors:** Talha Bin Emran, Md Atiar Rahman, Mir Muhammad Nasir Uddin, Raju Dash, Md Firoz Hossen, Mohammad Mohiuddin, Md Rashadul Alam

**Affiliations:** Department of Pharmacy, BGC Trust University Bangladesh, Chittagong, 4000 Bangladesh; Department of Biochemistry and Molecular Biology, University of Chittagong, Chittagong, 4331 Bangladesh; Department of Pharmacy, University of Chittagong, Chittagong, 4331 Bangladesh

**Keywords:** *Bacopa monnieri* L, *Staphylococcus aureus*, Antibacterial activity, MIC, Molecular docking, GOLD, *in silico* drug discovery

## Abstract

**Background:**

*Bacopa monnieri* Linn. (Plantaginaceae), a well-known medicinal plant, is widely used in traditional medicine system. It has long been used in gastrointestinal discomfort, skin diseases, epilepsy and analgesia. This research investigated the *in vitro* antimicrobial activity of *Bacopa monnieri* leaf extract against *Staphylococcus aureus* and the interaction of possible compounds involved in this antimicrobial action*.*

**Methods:**

Non-edible plant parts were extracted with ethanol and evaporated *in vacuo* to obtain the crude extract. A zone of inhibition studies and the minimum inhibitory concentration (MIC) of plant extracts were evaluated against clinical isolates by the microbroth dilution method. Docking study was performed to analyze and identify the interactions of possible antimicrobial compounds of *Bacopa monnieri* in the active site of penicillin binding protein and DNA gyrase through GOLD 4.12 software.

**Results:**

A zone of inhibition studies showed significant (*p* < 0.05) inhibition capacity of different concentrations of *Bacopa monnieri*’s extract against *Staphylococcus aureus*. The extract also displayed very remarkable minimum inhibitory concentrations (≥16 μg/ml) which was significant compared to that (≥75 μg/ml) of the reference antibiotic against the experimental strain *Staphylococcus aureus*. Docking studies recommended that luteolin, an existing phytochemical of *Bacopa monnieri,* has the highest fitness score and more specificity towards the DNA gyrase binding site rather than penicillin binding protein.

**Conclusions:**

*Bacopa monnieri* extract and its compound luteolin have a significant antimicrobial activity against *Staphylococcus aureus*. Molecular binding interaction of an *in silico* data demonstrated that luteolin has more specificity towards the DNA gyrase binding site and could be a potent antimicrobial compound.

## Background

Effective therapeutic options to combat *Staphylococcus aureus* infection are still limited. And this makes a major burden to control *Staphylococcus aureus* [1]. *S. aureus* is a commensal Gram-positive bacterium, which colonizes in human nasal mucosa either permanently or transiently [2], causing severe infections eventually [3,4]. But the clinical symptoms are not visualized until the immune system is affected [5]. However, the major problem in controlling the *S. aureus* infection is the occurrence of multi-drug resistance produced mainly due to the misuse of antibiotics. This is also caused by the treatment of non-bacterial infections with antibiotics or inadequate compliance with the regulations for drug ingestion. Therefore, new therapeutic molecule is an urgence to be introduced as antibiotic in the treatment of multi-drug resistant *S. aureus.* Several studies have proposed that phytocompounds are the best alternative to develop therapies for multidrug resistant bacterial infections [6-8].

*Bacopa monnieri* (L.) Wellst. (Family: Plantaginaceae) is known as *Herpestis monniera*. It is a water hyssop or “Brahmi” and is reputed as Ayurvedic medicine. It is used for gastrointestinal discomfort, rejuvenation, promoting memory and intellect, skin disorders, epilepsy, pyrexia and analgesia [9]. Number of biologically active compounds has been isolated from this plant. GC-MS analysis of the leaf extract of this plant showed the presence of tetracyclic triterpenoids, saponin, bacosides A and B phytosterols, hersaponin, d-mannitol, flavonoids viz., luteolin-7-glucoside, apigenin-7-glucocronide, alkaloids such as nicotine and herpestine, betulic acid, β-sitosterol, stigma-sterol and its esters, aspartic acid, glutamic acid and serine [10]. Despite enormous possibilities of this plant, no compound-activity relationship study has been conducted yet to investigate the phytochemicals responsible for its antimicrobial action. This research evaluates the *in vitro* antimicrobial activity of *B. monnieri* against *S. aureus* establishing the interaction of existing phytocompounds involved in this antimicrobial activity through *an in silico* molecular docking analysis [11,12].

## Methods

### Media and chemicals

Mueller-Hinton broth and agar media (Hi media, India; final pH 7.3 ± 0.2 at 25°C), was used for the determination of MIC and antibacterial activity. Tetracycline (50 μg/disk) and ampicillin disks (50 μg/disk) were procured from Oxoid, England.

### Collection and identification of plant materials

The plant *B. monnieri* was selected by Talha Bin Emran, Lecturer, Department of Pharmacy, BGC Trust University Bangladesh. Fresh leaves of *B. monnieri* were collected from the Chittagong University hilly forest on December 2013. The plant was identified by Dr. Shaikh Bokhtear Uddin, Taxonomist and Associate Professor, Department of Botany, University of Chittagong-4331, Bangladesh. A voucher specimen (Accession Number: 36285) containing the identification characteristics of the plant has been preserved in the Bangladesh National Herbarium for future reference.

### Preparation of crude ethanol extract

The fresh leaves of *B. monnieri* were washed immediately after collection and chopped into small pieces, air dried and ground (Moulinex Blender AK-241, Moulinex, France) into powder (40-80 mesh, 500 g). The resulting powder was soaked in an Erlenmeyer flask of absolute ethanol (2.0 L, at room temperature) and left for seven days allowing occasional stirring of the flask. Filtrate obtained through cheesecloth and Whatman filter paper No. 1 was concentrated under reduced pressure at the temperature below 50°C using a rotatory evaporator (RE 200, Bibby Sterling Ltd., UK). The extracts (yield 4.4 - 5.6% w/w) were all placed in glass Petri dishes (90 × 15 mm, Pyrex, Germany) to allow an air-dry for complete evaporation of solvent.

### Study of antibacterial activity

#### Bacterial strain

Gram-positive *Staphylococcus aureus* (ATCC6538) was used for screening the antibacterial effect of the plant extract. Bacterial strain was collected from the Microbiology Division of Bangladesh Council of Scientific and Industrial Research (BCSIR), Chittagong-4220, Bangladesh.

### Preparation of sample solutions

Small amount (1, 2 and 3 mg) of solid sample was dissolved in a definite volume (1 ml) of DMSO to make a solution of 1 mg/ml. DMSO was chosen as solvent because it does not have any inhibitory effect on bacterial cultures and it has extraordinary capacity to dissolve solid sample completely.

### Media preparation

The bacterial strain was grown and maintained on Standard Nutrient Agar (DIFCO) media (Hi media, India) at 37°C and pH 7.3 ± 0.2. The bacterium was sub-cultured overnight in nutrient agar broth which was further adjusted to obtain turbidity comparable to McFarland (0.5) standard when required. Test tube slants of nutrient agar medium were prepared for the maintenance of culture. Then a small amount of the collected microorganism was transferred to the test tubes with the help of sterilized needles. A number of test tubes were freshly cleaned for bacterial pathogen. The inoculated slants were inoculated at temperature below laboratory condition.

### Antibacterial screening through disk diffusion technique

The antibacterial activity of the extract was determined by disk diffusion technique (National Committee for Clinical Laboratory Standards, NCCLS, 2002). The test microbes were taken from the broth culture with inoculating loop and transferred to test tubes containing 5.0 ml sterile distilled water. The inoculums were added until the turbidity was equal to 0.5 McFarland standards. Cotton swab was then used to inoculate the test tube suspension onto the surface of the Muller Hinton agar plate and the uniformly swabbed plates were then allowed to dry. On the dry inoculated surfaces prepared paper disks were placed as follows. Sterilized Whatman paper disks (6 mm in diameter) were prepared previously by punching the filter paper with the help of a punch machine. After that the disks were placed upon 0.5 ml of the desired solution (1, 2 and 3 mg/disk) of the extract. After each application the disks were allowed to the temperature 40°C (one minute) for drying purposes. The disks containing plant extract were placed with blunt-nosed thumb forceps on the inoculated plates at equidistance in a circle. These plates were kept for 4-6 h at a low temperature (<8°C) to allow for diffusion of the extract from the disk into the medium. The same was done for negative control (ethanol). The plates were incubated at 37°C for 24 h. The experiment was conducted in triplicates. Antimicrobial activity was determined by a measurement of the inhibition zone diameter (mm) around each test organism.

### Minimum inhibitory concentration (MIC) determination

Minimum inhibitory concentration was determined by the microdilution method using serially diluted (2 folds) plant extract according to the National Committee for Clinical Laboratory Standards (NCCLS) (National Committee for Clinical Laboratory Standards, 2000). The MIC of the extract was determined by the dilution of *B. monnieri* extract with the concentrations of 0.0-25, 0.0-50, 0.0-75, 0.0-100, 0.0-125, and 0.0-150 μg/ml. Equal volume of each extract and nutrient broth was mixed in a test tube. Specifically 0.1 ml of standardized inoculum (1-2 × 10^7^ cfu/ml) was added in each tube. The tubes were incubated aerobically at 37°C for 18-24 h. Two control tubes were maintained for each test batch. These included antibiotic control (a tube containing extract and growth media without inoculum) and organism control (a tube containing the growth medium, saline and the inoculum). The lowest concentration (highest dilution) of the extract that produced no visible bacterial growth (no turbidity) was considered as MIC.

### Statistical analysis

All data are presented as mean ± standard deviation (SD). The data were analyzed by a statistical software statistical package for social science (SPSS, version 18.0, IBM Corporation, NY, USA) using Tukey’s multiple range *post hoc* tests. The values were considered significantly different at *p* < 0.05.

### Docking approach

**Figure 1:**
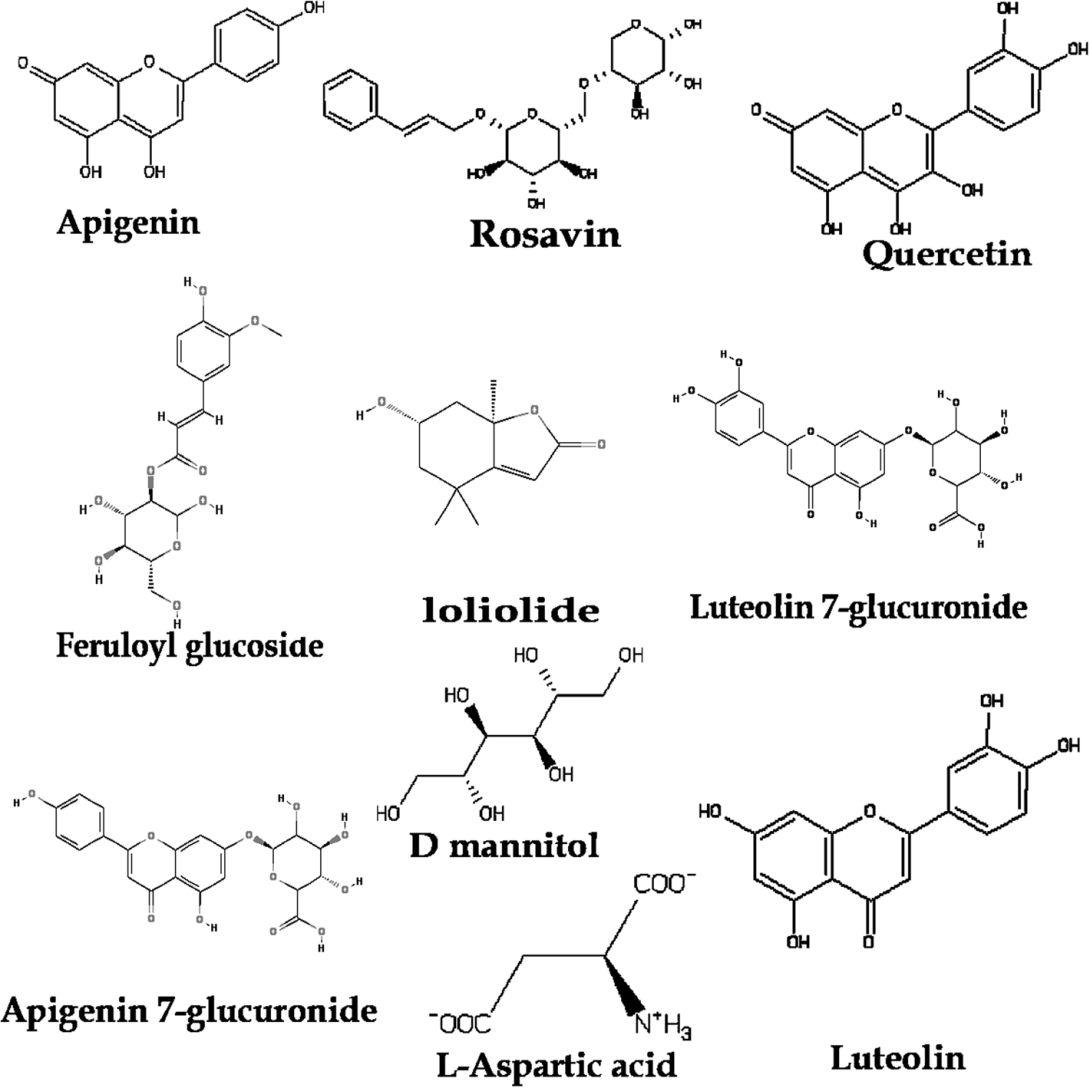
2D structure of all compounds of *B. monnieri.*

To have a better understanding about the inhibitory mechanism as well as the mode of interactions of the phytochemical compounds of the crude extract, docking analysis was accomplished using the GOLD 4.12 package. Two primary drug-target-pathways, i.e., penicillin-binding protein [13] and DNA gyrase [14] of *S. aureus* were subject to forecast the mechanism of plant derived compounds. Protein X-ray structure pdb ID: 3vsl and 3g7b was retrieved from protein data bank [15] and compared with standard inhibitor orientation in crystal structure. From the literature review, all compounds represented in Figure 1 were drawn in Symyx Draw 4.0 and to prepare for docking using the Sybyl 7.3 Molecular Modeling Suite of Tripos, Inc. Three dimensional (3D) conformations generated by using Concord 4.0 [16]; hydrogen atoms were added and charges were loaded using the Gasteiger and Marsili charge calculation method [17]. Basic amines were protonated and acidic carboxyl groups were de-protonated prior to charge calculation. The ligands were minimized with the Tripos Force Field prior to docking using the Powell method with an initial Simplex [18] optimization and 1000 interactions or gradient termination at 0.01 kcal/(mol*A). The input ligand file format was mol2 for all docking programs investigated. Three dimensional structure of standard drugs i.e., penicillin G and ciprofloxacin was occupied from zinc databases. The docking tool “GOLD” utilizes genetic algorithm to explore the rotational flexibility of receptor hydrogen’s and ligand conformational flexibility [19]. Such GOLD docking was carried out using the wizard with default parameters population size (100); selection pressure (1.1); number of operations (10,0 00); number of islands (1); niche size (2); and operator weights for migrate (0), mutate (100), and crossover (100). The active site with a 10 Å radius sphere was defined by selecting an active site residue of protein. Default genetic algorithm settings were used for all calculations and a set of 10 solutions was saved for each ligand. GOLD was used by a GoldScore fitness function. GoldScore is a molecular mechanism like function and has been optimized for the calculation of binding positions of ligand. It takes into account for four terms:

$$ \mathbf{Fitness}={\mathbf{S}}_{\left(\mathrm{h}\mathrm{b}\_\mathrm{e}\mathrm{x}\mathrm{t}\right)}+\mathbf{1.3750}*{\mathbf{S}}_{\left(\mathrm{v}\mathrm{d}\mathrm{w}\_\mathrm{e}\mathrm{x}\mathrm{t}\right)}+{\mathbf{S}}_{\left(\mathrm{h}\mathrm{b}\_\mathrm{i}\mathrm{n}\mathrm{t}\right)}+\mathbf{1.0000}*{\mathbf{S}}_{\left(\mathrm{i}\mathrm{n}\mathrm{t}\right)} $$

$$ {\mathbf{S}}_{\left(\mathrm{i}\mathrm{n}\mathrm{t}\right)}={\mathbf{S}}_{\left(\mathrm{v}\mathrm{d}\mathrm{w}\_\mathrm{i}\mathrm{n}\mathrm{t}\right)}+{\mathbf{S}}_{\left(\mathrm{tors}\right)} $$

Where, S _hb_ext_ is the protein-ligand hydrogen bonding and s _vdw_ext_ are the Vanderwaals interactions between protein and ligand. S _hb_int_ are the intramolecular hydrophobic interactions whereas S _vdw_ int_ is the contribution due to intramolecular strain in the ligand.

## Results

### *In vitro* antimicrobial assay

Results for the antibacterial activity of *B. monnieri* extract showed that the mean zone of inhibition (13.0-15.0 mm) produced by the extract was close to those produced by the reference antibiotics, i.e., tetracycline and ampicillin which had the zone of inhibitions between 16 to 20 mm. The extract of three different concentrations (1, 2 and 3 mg/disk) produced significant (*p* < 0.05) zone of inhibition against *S. aureus* and the values were 13.33 ± 2.08, 13.33 ± 2.08 and 15.33 ± 1.52 for 1, 2 and 3 mg/disk, respectively. The result of antibacterial activity of *B. monnieri* ethanol extract is shown in Table 1.

**Table 1:** ***in vitro*** **antibacterial activity of** ***B. monnieri*** **ethanol extract**

**Bacterial type**	**Test organism**	**Source ID (ATCC)**	**Diameter of zone of inhibition (mm)**				
			***Bacopa monnieri***			**Standard antibiotics**	
Gram + ve			1 mg/disk	2 mg/disk	3 mg/disk	Tetracycline (50 μg/disk)	Ampicillin (50 μg/disk)
	*Staphylococcus aureus*	6538	13.33 ± 2.08^a^	13.33 ± 2.08^b^	15.33 ± 1.52^c^	16.00 ± 3.54^d^	20.00 ± 1.60^e^

Data are shown as mean ± SD for triplicate of concentration. Different superscript letters (a-e) shown in the data indicate that the values are significantly different (Tukey’s multiple range, *post hoc* test, *p* < 0.05) from each other.

### Minimum inhibitory concentration

The minimum inhibitory concentrations of *B. monnieri* leaf extract for different bacterial strains were ranged from 25 to 100 μl/ml (Table 2). The arbitrary MIC against the Gram-positive bacteria *S. aureus* was greater than or equal to 75 for the extract and 16 for the reference antibiotic tetracycline.

**Table 2:** **Minimum inhibitory concentrations (MIC) of** ***B. monnieri*** **and tetracycline against** ***Staphylococcus aureus***

**Test organism**	**MIC of** ***Bacopa monnieri*** **extract (μg/ml)**	**MIC of tetracycline (μg/ml)**
*Staphylococcus aureus*	≥75	≥16

### Docking experiments

Considering the results obtained in *in vitro* study, it was thought worthy to perform molecular docking studies which correlate both *in silico* and *in vitro* results. Docking studies are used at different stages of drug discovery such as to predict a ligand-receptor interaction and also to rank the compounds based on the binding energies or fitness score [20]. In our present study, docking of tested compounds with the primary drug pathway for *S. aureus* was performed, and the corresponding fitness score was also determined as shown in Table 3. The interacting energies followed the order of the best fitness core. Highest fitness scored compound was further subjected to compare its binding pattern and molecular interaction with the standard drug penicillin G and ciprofloxacin.

**Table 3:** **Gold fitness score of** ***B. monnieri’s*** **all compounds against DNA gyrase and penicillin binding protein**

**DNA gyrase**						**Penicillin binding protein**				
**Compound name**	**Fitness score**	**S(hb_ext)**	**S(vdw_ext)**	**S(hb_int)**	**S(int)**	**Fitness Score**	**S(hb_ext)**	**S(vdw_ext)**	**S(hb_int)**	**S(int)**
Apigenin	46.59	5.57	36.00	0.00	-8.48	41.73	7.29	30.70	0.00	-7.77
Rosavin	51.58	5.44	42.31	0.00	-12.03	41.96	8.88	31.27	0.00	-9.92
Quercetin	45.71	6.04	37.32	0.00	-11.64	40.14	9.37	30.55	0.00	-11.23
Feruloyl glucoside	49.23	8.14	42.78	0.00	-17.74	32.42	4.76	35.88	0.00	-21.68
Loliolide	29.61	0.10	23.41	0.00	-2.68	31.02	3.34	22.10	0.00	-2.71
Luteolin-7-glucoside	47.87	10.04	42.46	0.00	-20.56	43.85	2.46	42.08	0.00	-16.48
Apigenin-7- glucocronide	45.63	8.34	39.19	0.00	-16.59	43.35	8.74	37.80	0.00	-17.36
D-mannitol	32.55	9.88	23.13	0.00	-9.13	30.21	8.15	20.48	0.00	-6.11
L-asperatic acid	33.35	17.74	17.07	0.00	-7.86	28.29	14.01	16.69	0.00	-8.66
Luteolin	53.77	11.23	37.44	0.00	-8.94	45.35	7.81	41.67	0.00	-19.77
Penicillin G	-------	-------	-------	-------	-------	46.48	2.62	35.14	0.00	-4.46
Ciprofloxacine	46.48	0.21	42.47	0.00	-8.94	--------	-------	-------	------	-------

**Figure 2:**
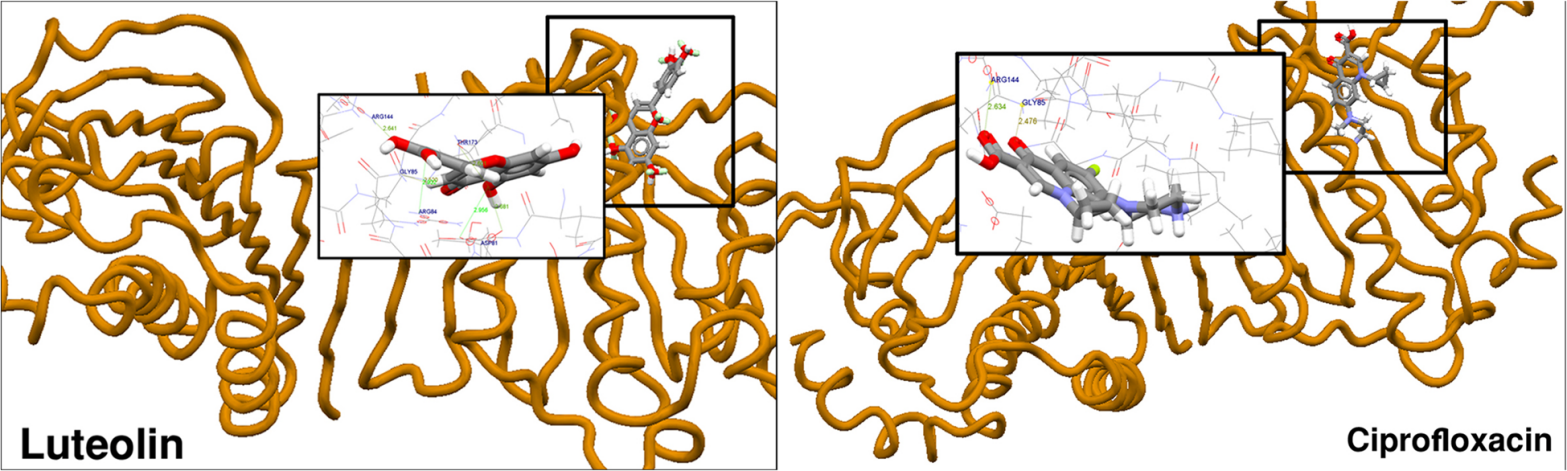
Interaction and superimposed structure of compound of luteolin and ciprofloxacin with DNA gyrase.

In the docking studies of DNA gyrase binding site, luteolin among the other tested compounds has the highest fitness score 53.77 compared with the highest fitness score 46.48 of ciprofloxacin. Molecular binding pattern of ciprofloxacin revealed that it has two hydrogen bonds with ARG144 and GLY85 consisting of hydrogen bonding distances 2.634 Å and 2.476 Å shown in Figure 2. These two hydrogen bonding residues in luteolin are found to be similar with different hydrogen bonds viz. 2.641 Å for ARG144 and 2.520 Å for GLY85. Additionally, it also formed two other hydrogen bonds with ARG84 and ASP81 having a bonding distance of 2.932 Å and 2.956 Å.

**Figure 3:**
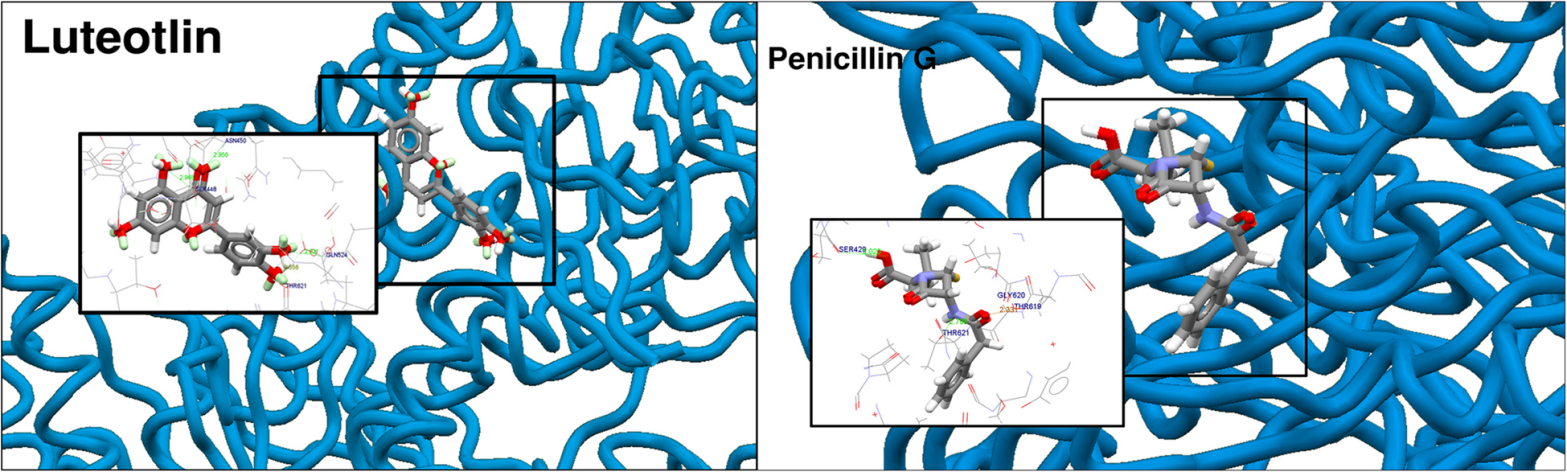
Interaction and superimposed structure of compound of luteolin and penicillin G with penicillin binding protein.

For the most potent inhibitor of penicillin binding protein, luteolin showed the highest fitness score among the other tested compounds viz., 45.35 fitness score compared to its reference standard drug penicillin G 46.48. In context of different binding patterns, luteolin has formed the three hydrogen bonds SER429, THR621 and THR619 where bonding distances were 3.020, 2.798 and 2.331. On the other hand, reference drug penicillin G formed hydrogen bonds with ASN450, SER778, THR621 and GLN524 with corresponding hydrogen bonds 2.956 Å, 2.968 Å, 2.556 Å and 3.031 Å, respectively. Binding mode and related interactions are summarized in Figure 3.

The emergence of bacterial resistance to current clinical drugs has brought intention to develop novel antimicrobial agents for selectively inhibiting the constantly evolved bacterial targets which have been also continually promoted with challenges. Presently known target of *Staphyloccus* sp. includes PBP (penicillin binding protein) of peptidoglycan biosynthesis pathway where beta-lactam antibiotics were known to be effective against it [21]. A different prescribing drug i.e. Fluroquinolone, DNA Gyrase A enzyme which is essential for the replication and super-coiling of DNA, is the main target at this case. But according to Stephen *et al*., a highly significant association between Levofloxacin and Ciprofloxacin treatment and consequent isolation of MRSA is reported [22]. However, in this research, molecular docking analysis suggested that luteolin has the more specificity towards the DNA gyrase binding site than penicillin binding protein. Regarding the obtained results, luteolin could serve as an appropriate starting point for designing new chemical entities as potent *S. aureus* inhibitor.

## Discussion

Plants have long been a very important source of drug and many plants have been screened whether they contain compounds with therapeutic activity. Therefore, it is vital to evaluate the antimicrobial activity of *B. monnieri*. The bacterial strain was chosen to be studied as it is an important pathogen and rapidly develop antibiotic resistance with its increased uses. In disk diffusion technique, the mean zone of inhibition produced by the commercial antibiotic, tetracycline and ampicillin, was larger than that produced by ethanol extract. It may be attributed to the fact that the plant extract being in crude form contains a smaller concentration of bioactive compounds. In classifying the antimicrobial activity it would be generally expected that a greater number would be active against Gram-positive than Gram-negative bacteria. Apart from this, the higher MIC value is an indication that either the plant extracts are less effective on bacteria or the organism has the potential to develop antibiotic resistance. On the contrary, the low MIC value for bacteria is an indication of the higher efficacy of the plant extracts.

Most of the pathogenic bacteria have developed resistance to currently available antibiotics due to their misuse or overuse. This situation has led to an urgent need to explore different sources of efficient, less toxic and cost-effective antimicrobial agents [23,24]. Medicinal plants play a major role and constitute the backbone of traditional medicine. According to the World Health Organization (WHO) estimate, 80% of populations in developing countries rely exclusively on traditional medicine for their healthcare need. Moreover, 20% of the available allopathic drugs have an active principal obtained from higher plants [25]. Recognizing the significance of indigenous medicinal plants WHO states in its 1997 guideline that locally available effective plants may be used as substitutes for drugs. Research work on medicinal plants and exchange of obtained information will go a long way in scientific exploration of medicinal plants for the benefit of mankind. This will ultimately decrease our dependence on synthetic drugs [26]. Plant synthesizes natural products as its chemical weapon that arrests the growth of environmental microbes [27] and some plants inhibit the growth of potential human pathogens too. In the current study, *in vitro* MIC of *B. monnieri* leaf parts, prescribed in indigenous system of medicine, that are available in the local market or growing in Bangladesh and India were evaluated against local clinical bacterial isolate of *S. aureus*. Determination of MIC of this plant is important to find out the best plant that eradicates infectious agents (Table 2). Clinicians also select the antibiotic on the basis of their MIC value to treat infectious diseases. Plant extracts having MIC below 8000 μg/ml have been reported as therapeutically effective [28]. Our results for *B. monnieri* implicated a significant MIC value (below or equal to 75 μg/ml) in this study. This significance suggests that we have identified antimicrobial activity of plant that is effective for arresting the growth of *S. aureus* causing hospital-,-acquired- and opportunistic- infections.

## Conclusions

*B. monnieri* extract and its compound luteolin have a significant antimicrobial activity against *S. aureus*. Molecular binding interaction of *in silico* data demonstrated that luteolin has more specificity towards the DNA gyrase binding site and could be a potent antimicrobial compound. However several scientific reports manifested that lead-drug discovery projects on the basis of binding efficiency indices would afford bioactive compounds with better pharmacokinetic outcomes. Hence, isolated bioactive compounds should be employed for establishing more rational structure activity relationships in the era of antimicrobial drug development.
